# Effect of Precursor Deficiency Induced Ca/P Ratio on Antibacterial and Osteoblast Adhesion Properties of Ag-Incorporated Hydroxyapatite: Reducing Ag Toxicity

**DOI:** 10.3390/ma14123158

**Published:** 2021-06-08

**Authors:** Ozkan Gokcekaya, Celaletdin Ergun, Thomas J. Webster, Abdurrahman Bahadir, Kyosuke Ueda, Takayuki Narushima, Takayoshi Nakano

**Affiliations:** 1 Division of Materials and Manufacturing Science, Osaka University, Suita 565-0871, Japan; ozkan@mat.eng.osaka-u.ac.jp (O.G.); nakano@mat.eng.osaka-u.ac.jp (T.N.); 2 Faculty of Mechanical Engineering, Istanbul Technical University, 34437 Istanbul, Turkey; bahadira83@gmail.com; 3 Department of Chemical Engineering, Northeastern University, Boston, MA 02115, USA; websterthomas02@gmail.com; 4 Department of Materials Processing, Tohoku University, Sendai 980-8579, Japan; ueda@material.tohoku.ac.jp (K.U.); narut@material.tohoku.ac.jp (T.N.)

**Keywords:** hydroxyapatite, silver, carbonate, incorporation, antibacterial, osteoblast adhesion

## Abstract

Ag-containing hydroxyapatite (HA) can reduce risks associated with bacterial infections which may eventually require additional surgical operations to retrieve a failed implant. The biological properties of HA in such applications are strongly affected by its composition in terms of dopants as well as Ca/P stoichiometry, which can be easily controlled by altering processing parameters, such as precursor concentrations. The objective of this in vitro study was to understand the effect of variations in HA precursor solutions on antibacterial properties against *Escherichia coli* *(E. coli)* and for promoting osteoblast (bone-forming cell) adhesion on Ag incorporated HA (AgHA) which has not yet been investigated. For this, two groups of AgHAs were synthesized via a precipitation method by adjusting precursor reactants with a stoichiometric value of 1.67, being either (Ca + Ag)/P (Ca-deficient) or Ca/(P + Ag) (P-deficient), and were characterized by XRD, FTIR, and SEM-EDS. Results showed that Ag^+^ incorporated into the Ca^2+^ sites was associated with a corresponding OH^−^ vacancy. Additional incorporation of CO_3_^2−^ into PO_4_^3−^ sites occurred specifically for the P-deficient AgHAs. While antibacterial properties increased, osteoblast adhesion decreased with increasing Ag content for the Ca-deficient AgHAs, as anticipated. In contrast, significant antibacterial properties with good osteoblast behavior were observed on the P-deficient AgHAs even with a lower Ag content, owing to carbonated HA. Thus, this showed that by synthesizing AgHA using P-deficient precursors with carbonate substitution, one can keep the antibacterial properties of Ag in HA while reducing its toxic effect on osteoblasts.

## 1. Introduction

Hydroxyapatite (HA, Ca_10_(PO_4_)_6_(OH)_2_), one of the main phases of calcium phosphate (CaP), has been investigated as a major mineral component of calcified tissues for several decades [1]. It has been used for various applications in medical applications, such as a drug carrier with controlled release, bone cement, toothpaste additive, or as a coating on metallic implants, for decades [2]. On the other hand, despite its excellent biocompatibility and osteoconductive properties when used as a biomaterial, HA is susceptible to bacterial infections [3]. HA was never designed to reduce bacteria function by itself.

Many biomaterial failures are associated with infection, which grows and can be disruptive to the natural healing process, eventually causing complete implant failure as a result of the development of microbial colonies and biofilms on an implant surface [4,5]. Infections caused by these organisms are usually treated by traditional systematic drug (antibiotic) administration. These common treatments have numerous drawbacks, such as possible toxicity to mammalian cells as well as low drug penetration into the target tissue [5,6]. Furthermore, there is now a global healthcare crisis in antibiotic-resistant bacteria, which are bacteria that have formed a resistance to antibiotics and can no longer be killed by such antibiotics (such as methicillin-resistant *Staphylococcus aureus* or MRSA). As an alternative, silver (Ag), as one of the oldest known antibacterial materials, has been used for wound disinfection and also in the treatment of numerous microbial infections [6,7].

However, the antimicrobial action of Ag has not been clearly understood yet, but it is assumed to be facilitated by the release of Ag^+^ to form a strong bond with electron donor groups in biological molecules. Nevertheless, two bacterial mechanisms have been proposed. In the first mechanism, Ag^+^ can first form pits on the bacteria membrane through oxidative reactions and then penetrate the cytoplasm eventually causing cell death [8,9], known as “contact killing”. In the second mechanism, Ag^+^ can bind to the microbial DNA in bacteria, preventing bacterial replication, and interacting with sulfhydryl groups on metabolic enzymes of the bacterial electron transport chain [10,11] known as “leach killing”. Unfortunately, however, the toxicity of Ag against mammalian cells is still a matter of concern particularly in their utilization as an antimicrobial agent in the human body. It is clear that to take advantage of the antibacterial properties of Ag, how it is incorporated into biomaterials needs to be carefully assessed. As provided by the highly flexible nature of the apatite structure, numerous researchers have tailored apatite properties via ionic substitutions into both cation (Ca^2+^) and anion (PO_4_^3−^ and OH^−^) sites in its structure [12]. In this respect, Ag incorporated HA (AgHA) has been widely studied to provide antimicrobial activity while maintaining the bioactivity of implants, providing bacterial inhibition, and enhancing osteoblast functions [13,14,15].

Thus, Ag can indeed be used as an implant material to prevent biomaterial-related post-implantation infections, a substantial problem affecting the long-term in vivo performance of implants, possibly leading to surgery to remove the failed implant in order to save a patient’s life [16,17]. However, the cytotoxicity of released Ag^+^ from AgHA still needs to be addressed as one of the main questions to determine the optimal Ag concentration to ensure both effectiveness against bacteria at the implant surface and safeness against the cytotoxic effect on nearby cells [18]. The vast majority of the studies focused on optimizing the amount of Ag to obtain antibacterial properties while maintaining mammalian cell activities [6,7,8,9,10,11,12,13,14,15]. However, this study focused on altering the HA structure with a precursor deficiency approach for the first time ever in the literature (to the best of the authors’ knowledge).

Chemical precipitation is a simple and cost-effective process for synthesizing AgHA particles [19]. Based on this process, the physicochemical properties of the synthesized particles are mainly controlled by several process parameters, i.e., temperature, pH, and the concentration of the precursors in an aqueous medium. During the synthesis of AgHA, the starting reactants are added into precursor solutions in appropriate molar proportions based on the desired final stoichiometry. In some research, assuming Ag^+^ incorporation into Ca positions, a certain amount of Ca precursor chemicals were replaced with Ag precursor chemicals on a molar basis to counterbalance the resulting Ca-deficient precursor and maintain the cation (Ca + Ag)/P atomic ratio at 1.67 [20]. In some other HA materials, P precursor chemicals were replaced with a dopant precursor chemical to counterbalance the resulting P-deficient precursor and maintain the cation Ca/(P + dopant) atomic ratio at 1.67 [21], however, this assumption has not been applied in terms of Ag^+^ incorporation. Even yet, in some other research, the reactants as dopant sources were directly added to precursor solutions even without any compensation to maintain the respected atomic ratio at 1.67, which can be defined as an extra precursor [22,23,24,25].

In fact, the solid solubility of Ag ions in the HA structure, in terms of the Ag/(Ca + Ag) atomic ratio, has been reported to be in the range of 0.0019–0.0061 [20]. In this solid solution, Ag incorporation into the Ca-site is suggested to be highly probable and the amount of Ag can be as much as an Ag/(Ca + Ag) atomic ratio of 0.0909 [26,27]. On the other hand, a small amount of Ag incorporation into the P-site is suggested to be possible with a Ag amount corresponding to an Ag/(Ca + Ag) atomic ratio of 0.016 [26].

Obviously, the difference in the precursor preparation to replace some Ca ions or P ions with Ag ions is based on the assumption that Ag will incorporate into Ca or P sites, and will energetically favor a particular resultant phase/s, eventually, determining the final properties of the incorporated HA. As a result, there is a bias to choose one of these approaches regarding Ca-deficiency or P-deficiency to optimize biological or other physicochemical properties for AgHA.

In this sense, the biological response of AgHAs should be evaluated depending on the Ag content as the characteristic of the precursor chemistry [26], to precisely tailor its final properties and to make correct predictions about its performance in real medical applications. With this motivation in mind, to the best of the authors’ knowledge, this in vitro study was conducted for the first time to systematically elucidate the effect of the stoichiometric value of 1.67 obtained via either (Ca + Ag)/P (Ca-deficient) or Ca/(P + Ag) (P-deficient) atomic ratios in precursor solutions (possessing 0.2 and 0.5 mol Ag addition) on antibacterial properties and osteoblast (bone-forming cell) adhesion. In doing so, this study provides a new way to synthesize AgHA to retain the antibacterial properties of Ag with reduced toxicity to osteoblasts.

## 2. Materials and Methods

### 2.1. Synthesis and Characterization

Pure HA and AgHAs were synthesized according to a well-established precipitation method [28]. Briefly, for pure HA synthesis, calcium nitrate (Ca(NO_3_)_2_·4H_2_O (Alfa Aesar, Karlsruhe, Germany; 99.99% purity)) and ammonium phosphate (NH_4_)_3_PO_4_ (Alfa Aesar, Karlsruhe, Germany; 99.99% purity)) solutions were separately brought to a pH of 11–12 with aqueous ammonia (NH_4_OH, 25% (Alfa Aesar, Karlsruhe, Germany; 99.99% purity)). The 1 M Ca(NO_3_)_2_·4H_2_O solution was stirred vigorously at room temperature (RT), and the 0.6 M (NH_4_)_3_PO_4_ solution was added dropwise into this Ca(NO_3_)_2_·4H_2_O solution to produce a gelatinous precipitate. The precipitated solution was stirred for 24 h at RT to form pure HA powders.

In order to synthesize AgHA [29], reagent grade silver nitrate (AgNO_3_ (Alfa Aesar, Karlsruhe, Germany; 99.99% purity)) was added into the Ca(NO_3_)_2_·4H_2_O solution. Ag incorporation into Ca or P ions was chosen at either 0.2 mol or 0.5 mol. There was a goal to achieve a stoichiometric value of 1.67 for the (Ca + Ag)/P or Ca/(P + Ag) atomic ratios, therefore, the atomic ratios of Ca or P ions were deliberately added at 0.2 mol or 0.5 mol less than that required for pure HA into the Ca deficient (C2 and C5) or P deficient (P2 and P5) precursor solutions, respectively, to compensate for the difference with Ag ions.

Additionally, samples were prepared for comparison purposes with the addition of 0.2 mol Ag (E2) to stoichiometric HA precursor solutions without any compensation in either Ca or P precursors. The description and the composition of the samples are shown in Table 1.

The reaction mixture was centrifuged and washed repeatedly with deionized water to remove the unreacted ionic species and ammonia solution. Next, the as-precipitated powders were filtered using a 0.2-µm filter paper, and then dried for at least 48 h at 60 °C. Dried powders were crushed and passed through a 200-mesh screen to obtain a powder with particle sizes <75 µm in diameter.

0.5 g of pure HA and AgHA powders were cold-pressed into 10 mm diameter cylindrical pellets under 100MPa pressure. Subsequently, the pellets were heat-treated at 700 °C, 900 °C, 1100 °C, and 1300 °C (ramp rate of 22 °C/min) for 2 h. The phases of the heat-treated samples were confirmed by X-ray diffraction (XRD, Philips PW2273/20, Kyoto, Japan).

The chemical composition of AgHAs was measured by scanning electron microscopy (SEM, JEOL JSM-840, Tokyo, Japan) equipped with energy-dispersive X-ray spectroscopy (EDS). The measured composition values were standardized with commercial calcium pyrophosphate (CPP, Ca/P = 1.0), tricalcium phosphate (TCP, Ca/P = 1.5), HA (Ca/P = 1.67), and tetra calcium phosphate (TTCP, Ca/P = 2.0). Fourier transform infrared (FTIR, JASCO, FT/IR-460Plus, Tokyo, Japan) spectroscopy was used to identify the functional groups and the presence of bonds formed in the heat-treated AgHAs. The model HA crystal structure was visualized with VESTA [30], according to the FTIR observations, and was proposed for AgHAs regarding Ca-deficiency or P-deficiency.

Grain size of the heat-treated pure HA and AgHAs was investigated by SEM. The densities of the cylindrical samples were calculated from their measured dimensions and mass. A parameter defined as the densification factor (*DF*) was used to monitor the density changes of AgHAs during the heat-treatment at different temperatures. This factor was calculated with respect to ref. [31] with the following equation:

(1) $$DF=\frac{D-D_{g}}{D_{g}}\times 100$$

where *D* is the density of the heat-treated samples and *D_g_* is the density of the cold-press powders.

### 2.2. In Vitro Evaluation

Only the pure HA and AgHAs heat-treated at 1100 °C were subjected to in vitro experiments to evaluate the effect of Ag incorporation into the HA structure on the respective biological behavior. Specifically, the bactericidal activity of AgHAs in the nutrient broth solution (NB, [Cl^−^] = 100 mM) was evaluated with the turbidimetric method. The details of this method were described elsewhere [15]. Briefly, the samples were placed in polypropylene (PP) test tubes and autoclaved at 121 °C under steam pressure for 21 min. Subsequently, they were exposed to 5 mL of an *Escherichia coli* (*E. coli*, DH5α) containing NB solution for 6 h while being shaken at 200 rpm and incubated at 37 °C, in which the control group reached 8 × 10^8^ colony-forming units (CFU)·mL^−1^. The tests were conducted with five heat-treated pellets with triplicate measurements from each sample.

Bacterial cultures without any powder were also incubated and used as a control group. After incubation, optical densities (*OD*) of the control and AgHA containing NB solutions were measured at 600 nm using a UV–vis spectrophotometer (Shimadzu BioSpec-1600, Kyoto, Japan).

A parameter defined as the bactericidal ratio was used to compare and evaluate the bactericidal properties of the samples. This ratio was calculated with the following equation [15]:

(2) $$\mathrm{Bactericidal}\;\mathrm{ratio}\;\left(\%\right)=\frac{\mathrm{OD}\;\mathrm{of}\;\mathrm{control}\;\mathrm{group}\;-\;\mathrm{OD}\;\mathrm{of}\;\mathrm{experimental}\;\mathrm{group}}{\mathrm{OD}\;\mathrm{of}\;\mathrm{control}\;\mathrm{group}}\times 100$$

Human osteoblasts (bone-forming cells; CRL-11372; ATCC) were cultured in Dulbecco’s modified Eagle’s medium (DMEM; GIBCO) supplemented with 10% fetal bovine serum (FBS; Hyclone) and 1% penicillin/streptomycin (P/S; Hyclone) under standard cell culture conditions (37 °C, humidified, 5% CO_2_/95% air environment). All heat-treated pellets were sterilized and placed in 12-well tissue culture plates and were rinsed three times with sterilized phosphate-buffered saline (PBS). Osteoblasts were seeded at a concentration of 2500 cells·cm^−2^ onto the samples of interest in DMEM supplemented with 10% FBS and 1% P/S and were then incubated under standard cell culture conditions for 4 h [32]. After incubation, non-adherent cells were removed by rinsing with PBS, and adherent cells were then fixed with formaldehyde (Fisher Scientific, Hampton, NH, USA) and stained with Hoechst 33258 dye (Sigma Aldrich, St. Louis, MD, USA); the cell nuclei were, thus, visualized and counted under a fluorescence microscope. Cell counts were expressed as the average number of cells on eight random places per sample.

Borosilicate glass coverslips (reference material) were etched in 1N of NaOH according to a standard protocol and used as a reference material in the experiments [32]. Additionally, pure HA was used as another reference material in the experiment to highlight the effect of the incorporated Ag ions. Osteoblast counts were expressed as the average number of cells on eight random places per sample.

### 2.3. Statistical Analysis

All in vitro experiments were carried out with five pellets, each sample in triplicate, and cell adhesion was evaluated based on the mean number of adherent cells while the bactericidal ratio was determined with *OD* values. Numerical data from the in vitro antibacterial and osteoblast adhesion experiments were analyzed using standard analysis of variance (ANOVA) techniques and statistical significance was considered at *p* < 0.05.

## 3. Results

### 3.1. Material Characterization

According to the spectral analysis (Table 2), pure HA had a Ca/P atomic ratio of 1.69 which is quite comparable to the ideal stoichiometric value of 1.67. However, E2 exhibited the highest Ca/P atomic ratio (2.06), indicating a possible P depletion from the HA structure. C2, C5, P2, and P5 had standardized Ca/P atomic ratios of 1.99, 1.81, 1.91, and 1.99, respectively. Moreover, C2 and C5 possessed Ag/(Ca + Ag) atomic ratios of 0.0025 and 0.0061 respectively. Furthermore, P2 and P5 possessed Ag/(Ca + Ag) atomic ratios of 0.0017 and 0.0050, respectively, which correlated well with the previous report [19]. These results revealed that the Ca/P atomic ratio decreased with increased Ag content in the Ca-deficient AgHAs, and, in contrast, an increase in P-deficient AgHAs.

On the other hand, E2 had a relatively lower Ag content compared to its counterparts which presumably seemed to be due to the inability of Ag ions in the precursor solutions to incorporate into the HA structure during the precipitation. The Ag cations might have reacted with P ions in the precursor solution possibly forming Ag_3_PO_4_ precipitates as reported in [20] during the synthesis. Therefore, E2 may have a higher Ca/P atomic ratio (Table 2).

**Figure 1:**
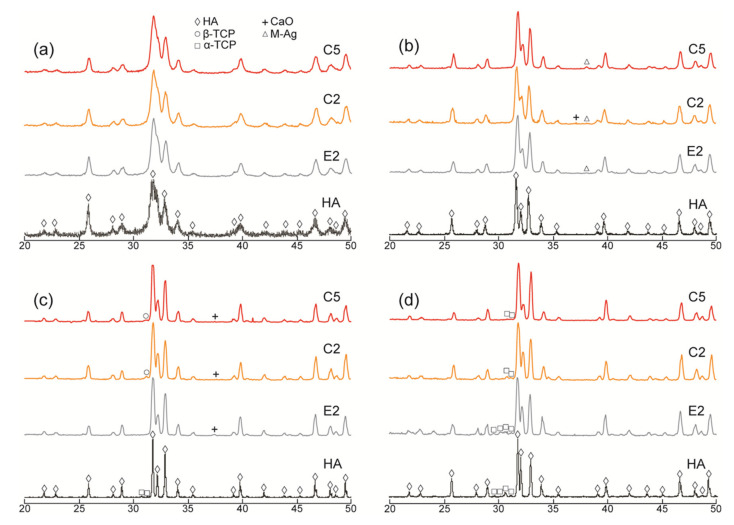
XRD patterns of the Ca-deficient (C2 and C5) AgHAs compared to E2 and pure HA heat-treated at (**a**) 700 °C, (**b**) 900 °C, (**c**) 1100 °C, and (**d**) 1300 °C. Y-axis: arbitrary unit.

**Figure 2:**
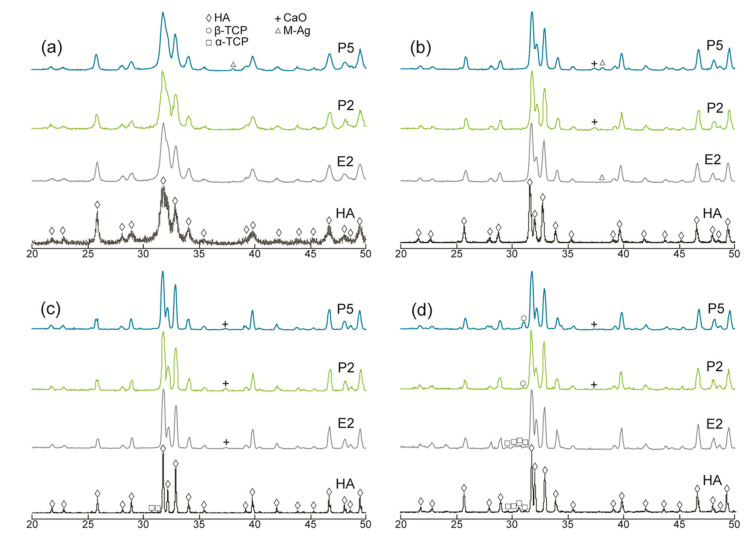
XRD patterns of the P-deficient AgHAs compared to E2 and pure HA heat-treated at (**a**) 700 °C, (**b**) 900 °C, (**c**) 1100 °C, and (**d**) 1300 °C. Y-axis: arbitrary unit.

XRD patterns of the pure HA and AgHAs heat-treated at 700 °C, 900 °C, 1100 °C, and 1300 °C are presented in Figure 1 and Figure 2, respectively. All patterns (being closely matched to JCPDS#9-0432) revealed that HA was the main phase in all samples. However, as indicated by the relatively broader peaks, it was partially crystallized at 700 °C and almost fully crystallized at 900 °C for all samples. The crystallinity of the HA phase seems to be more dominant in the pure HA than in the AgHAs at all temperatures, which may be due to the smaller particle size and/or partial disorderliness, most probably resulting from Ag incorporation into the HA structure as well as the corresponding formation of the other structural defects.

In the meantime, a slight amount of metallic Ag, as a minor phase, was detected in all AgHAs at 900 °C (Figure 1 and Figure 2), except P2 perhaps due to its relatively lower Ag content (Table 2). Furthermore, the metallic Ag seemed to disappear at 1100 °C, most probably due to incorporation into the HA and/or β-TCP phases with possible solid-state diffusion [26].

α-TCP was observed only in pure HA at 1100 °C as a result of the partial decomposition of the HA phase. However, a slight amount of β-TCP was observed in Ca-deficient AgHAs at 1100 °C, while it transformed into α-TCP at 1300 °C (Figure 1c). On the contrary, P-deficient AgHAs showed no apparent decomposition of HA at 1100 °C (Figure 2c), whereas, they showed a partial decomposition of HA, forming only a slight amount of β-TCP at 1300 °C (Figure 2d).

**Figure 3:**
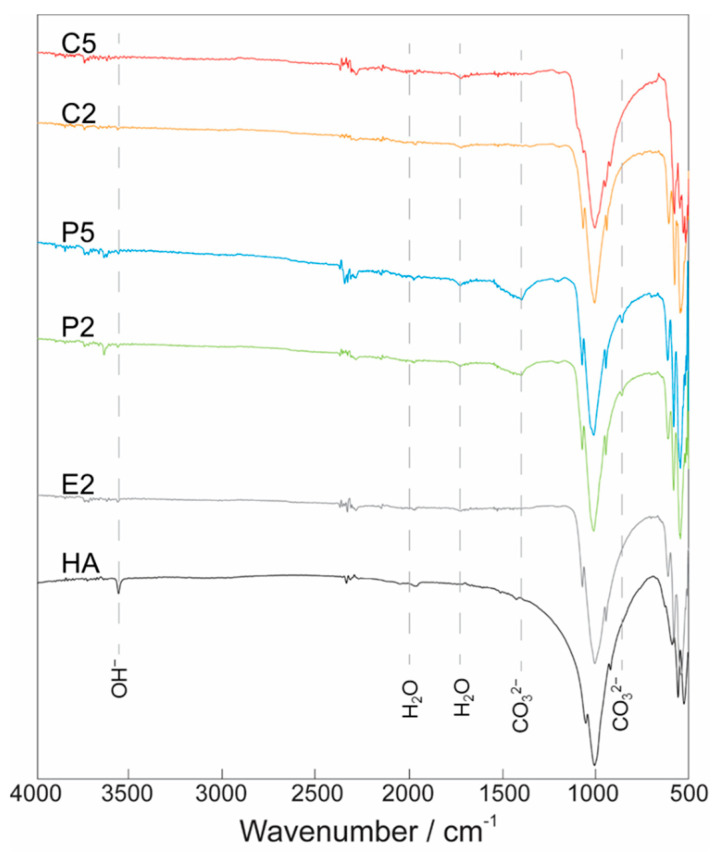
FTIR spectra for Ca-deficient (C2 and C5) and P-deficient (P2 and P5) AgHAs compared to E2 and pure HA heat-treated at 1100 °C. Y-axis: arbitrary unit.

The FTIR spectra of HA and AgHAs illustrated in Figure 3 showed systematic changes in phosphate, hydroxyl, and carbonate bands as a response to the changing sample type and increasing Ag content. For instance, the strong peaks in the range 900–1200 cm^−1^ attributed to P−O stretching vibration modes of phosphate groups showed a decrease in intensity with an increase in Ag content. On the other hand, the peak observed at 1630 cm^−1^ assigned to the vibration band of the lattice of water did not show any significant change.

The peaks observed in the range of 1400−1500 cm^−1^ and 885 cm^−1^ in the P2 and P5 samples can be attributed to the asymmetric stretching vibration of CO_3_^2−^, particularly corresponding to B-type carbonate groups that specifically incorporated into the phosphate sites [33]. In contrast, no peak was identified as evidence for the existence of A-type carbonate [34]. In the meantime, the peak of the band at 3570 cm^−1^ attributed to the stretching vibration of OH^−^ in the HA structure almost completely disappeared from the FTIR spectra of E2, P2, P5, C2, and C5 (Figure 3).

**Figure 4:**
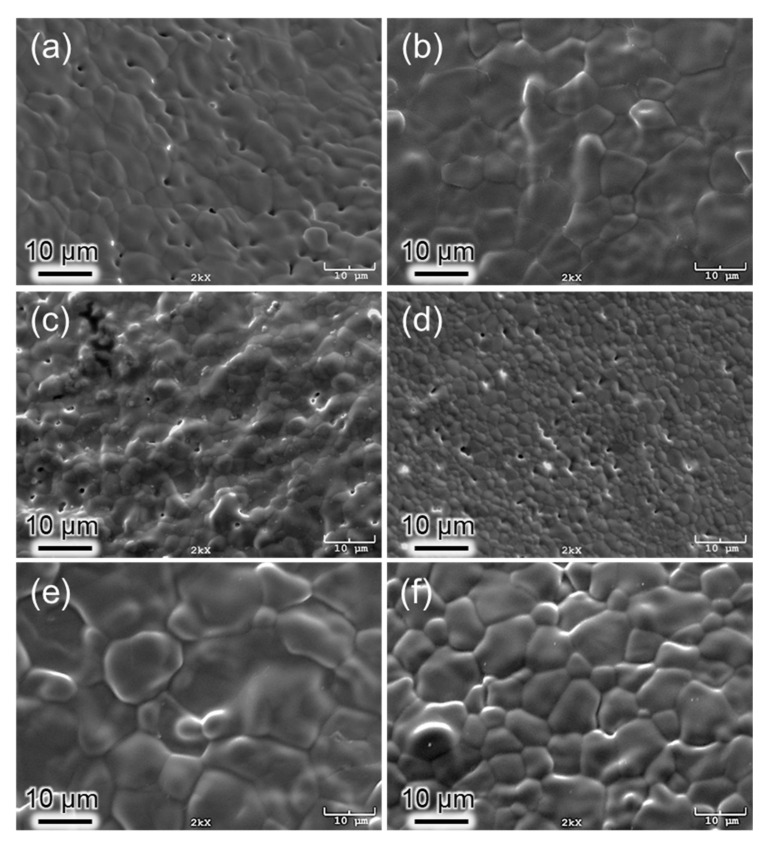
SEM images of AgHAs and pure HA heat-treated at 1100 °C: (**a**) HA, (**b**) E2, (**c**) C2, (**d**) C5, (**e**) P2, and (**f**) P5. (Scale bar = 10 µm).

The results from the SEM studies (Figure 4) showed that HA, C2, and C5 possessed a small amount of porosity at 1100 °C, providing evidence for the possible thermal decomposition of the HA main phase, as indicated by the XRD results. The average grain size of these samples was 5.8 µm, 5.3 µm, and 3.3 µm, respectively (Table 2). However, the average grain sizes of E2, P2, and P5, showing no phase decomposition, were 8.6 µm, 10.6 µm, and 6.6 µm, respectively.

### 3.2. Densification

**Figure 5:**
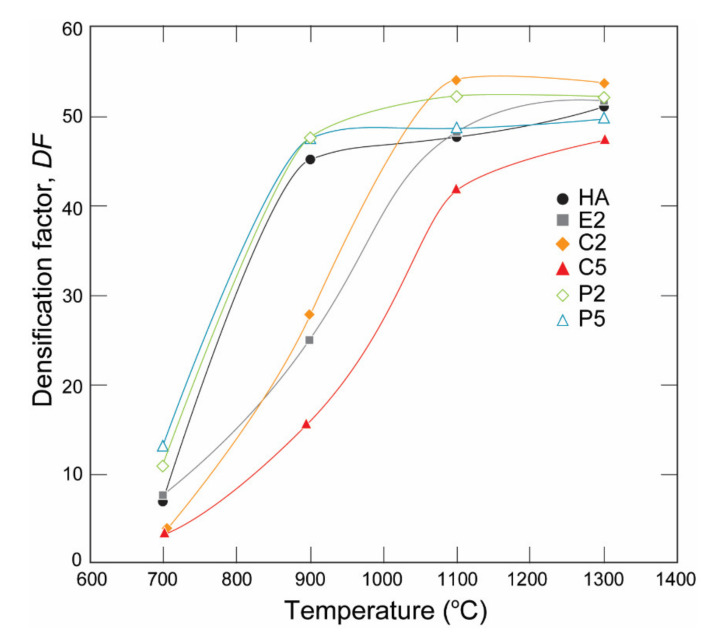
Densification factor of the HA, C-Ag and P-AgHA series.

The densification factor (DF) of the samples calculated using Equation (1) is given in Figure 5. The DF values of the samples showed a steep increase with an increase in the heat-treatment temperature from 700 °C to 1100 °C. Apparently, the increase in densification occurred earlier, particularly in P2, and the P5 samples at a lower temperature range and later for E2, C2, and C5 at a higher temperature range. The *DF* of C2 was the highest among the samples at 1100 °C and 1300 °C. In contrast, the *DF* of C5 was always the smallest at all temperatures, which can be related to the dehydration of the OH^−^ group [31] and a subsequent phase decomposition during the heat treatment detected with XRD analysis, which releases H_2_O as a reaction product as discussed by Ou et al. [35] and resulted in differences in the thermal expansion of HA and β-TCP [36]. This result can be related to the porosities observed in the SEM micrograph (Figure 4).

### 3.3. Bactericidal Effect

**Figure 6:**
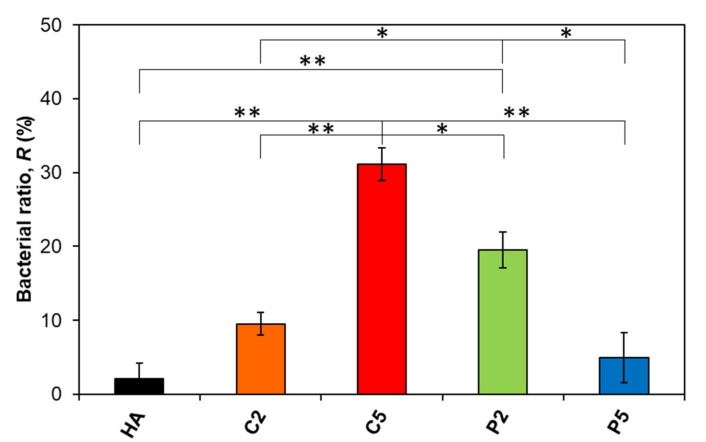
Change in the bactericidal ratio of the AgHA series. (** *p* < 0.01, * *p* < 0.05).

The bactericidal ratios of the samples of interest in this study as calculated with Equation (2) are given in Figure 6. The descending order in bactericidal ratios from this study was C5 > P2 > C2 > P5 > HA, while the control group reached 8 × 10^8^ CFU mL^−1^ bacteria concentration after 6 h of incubation, which is quite a high bacteria concentration considering that clinical infection occurs at about a 1 × 10^5^ CFU mL^−1^ bacteria concentration.

The ratio was 2.1% for pure HA, which was the minimum value among the samples. On the other hand, the ratio increased to 9.5% for C2 and further increased to 31.1% for C5. These results seem to be quite plausible since C5 had a higher Ag content than C2 (Table 2). In contrast, the ratio was approximately 20% for P2 which is higher than that for C2 despite its relatively lower Ag content. Interestingly, the ratio further decreased to 4.9% for P5 despite an even higher Ag content than both P2 and C2 (Table 2). As a result, increasing the Ag content increased the bactericidal ratio in the Ca-deficient AgHAs, which in contrast, decreased in the P-deficient ones. Moreover, P2 exhibited a significant difference in bactericidal ratio compared to HA, even C2 and P5 with higher Ag content.

### 3.4. Osteoblast Adhesion

**Figure 7:**
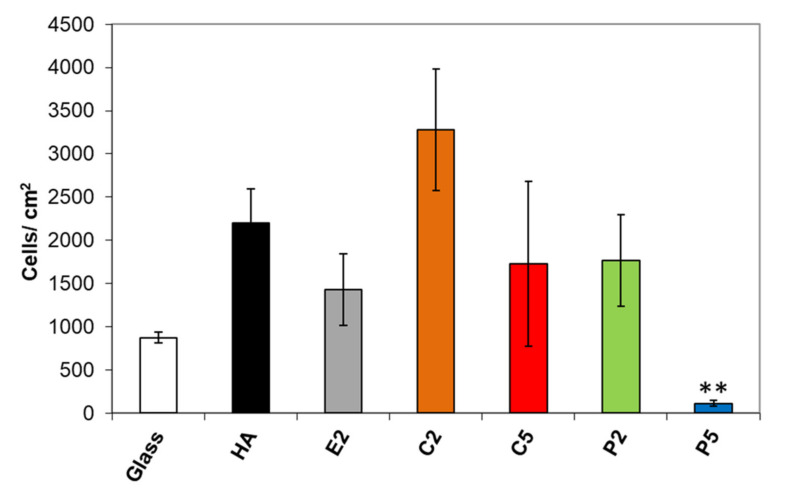
Change in osteoblast adhesion on the AgHA series. (** *p* < 0.01; compared to HA).

Results of osteoblast adhesion to the samples after 4 h are shown in Figure 7. The difference in osteoblast adhesion on the AgHA was statistically insignificant except for P5 compared to that on pure HA. The mean value of viable osteoblasts was the highest for C2, being better than pure HA with a factor of 1.5. In contrast, osteoblast adhesion exhibited a considerable decrease for C5, which is not surprising since it had a higher Ag content than C2. On the other hand, the mean value of viable osteoblasts on the P2 sample was interestingly lower than on C2 despite its lower Ag content. More interestingly, the number of viable cells decreased on the P5 samples, being a much lower value than that on C5 with a factor of about 15.6, in spite of its slightly lower Ag content.

## 4. Discussion

### 4.1. The Effect of a Deficient Precursor Approach on the AgHA Structure

The AgHAs synthesized from the Ca-deficient precursors, namely C2 and C5, showed a partial decomposition in the HA phase forming a slight amount of β-TCP upon heat treatment at 1100 °C [13]. In contrast, the AgHAs synthesized from the P-deficient precursors, namely P2 and P5, stayed mainly stable at 1100 °C, while a slight amount of β-TCP became apparent at 1300 °C. For comparison, HA and E2 were considered, which presented a similar structural decomposition to α-TCP at 1300 °C which is higher than the β→α transformation temperature (around 1140 °C). In this regard, the Ag ion can be considered as a β-TCP stabilizing agent upon incorporation into the HA structure [20], especially for the P-deficient precursors in this study.

In order to compare the Ag content in AgHAs for the heat-treated conditions, a parameter called the concentration factor $\left(\mathrm{CF}=\frac{\mathrm{Ag}}{\mathrm{Ag}\;+\;\mathrm{Ca}\;+\;P}\right)$, was calculated based on the results of the elemental analysis (Table 2). The CFs for C2 and C5 were 0.0017 and 0.0042, respectively. On the other hand, the respective values for P2 and P5 were 0.0011 and 0.0035, respectively. Therefore, it seems that a higher amount of Ag incorporation became possible when synthesized from the Ca-deficient precursors. Upon considering the estimated solubility limit of Ag (described as the Ag/(Ca + Ag) atomic ratio) being in the range of 0.0019–0.0061 [20], the Ag content in C5 reached the highest in this study at 0.0061, which seemed to be in a good agreement with the literature [20,26,37]. However, E2 showed a considerably lower Ag content (0.0004 atomic ratio), highlighting the effectiveness of using deficient precursor approaches in successful Ag incorporation in the HA structure. Therefore, the lack of Ag incorporation in E2 and a high Ca/P atomic ratio was predicted to be the result of possible Ag_3_PO_4_ formation during the precipitation process as mentioned in Section 3.1. Thus, further discussion here is mainly focused on the synthesis by Ca-deficient and P-deficient precursors.

FTIR results showed that the P–O stretching bands (900−1200 cm^−1^) became weaker upon Ag incorporation as observed in all AgHAs compared to the pure HA. Indeed, as reported in the literature, the incorporation of larger cations, such as Sr^2+^, into Ca sites is expected to cause weakening in the P–O stretching bands because of the formation of a subsequent larger anion–anion separation, while the incorporation of smaller cations, such as Mg^2+^, is expected to show strengthening in the P–O stretching bands because of the smaller anion–anion separation that it causes [38]. In this regard, it is plausible to assume that the incorporation of larger Ag^+^ cations into smaller Ca^2+^ sites can lead to an expansion in the lattice parameters [20] causing a larger anion–anion separation, as observed in this study. Nevertheless, this assumption by itself just considers that the ion sizes would not be sufficient to understand the incorporation mechanism since the charge of the incorporated ions and the strength of the corresponding established bonds should also play an important role in determining energetically preferable sites, particularly when monovalent cation, such as Ag^+^, incorporation takes place. For instance, even though the incorporation of divalent cations is not supposed to cause any charge imbalance in the HA lattice, incorporation of monovalent cations, such as Ag^+^, can cause an overall charge imbalance that can be neutralized simply by vacancy formation [39], or complex multi-ion incorporation with a combination of various cations and anions preserving an overall charge balance with or without additional vacancy formation [40].

In this regard, the absence of peaks for OH^−^ groups in the FTIR spectra of AgHAs can suggest that monovalent Ag^+^ ions were incorporated into the divalent Ca(1) positions coupled with the subsequent formation of an OH^−^ vacancy to preserve the overall charge balance. Ag^+^ ions can preferably be substituted, particularly into Ca(1) sites, because not only are Ag^+^ ions larger, but also these sites have coordination with OH^−^ with a relatively weaker bond and therefore the formation of this defect couple may be energetically more favorable [33]. Thereby, weaker P–O stretching bands seem to be observed in the AgHA samples with an increase in Ag content (Figure 3) due to the subsequent increase in anion–anion separation [41].

With this in regard, the following substitution model for the Ca deficient samples can be proposed:

(3) $${\mathrm{Ca}}_{10-x}{\mathrm{Ag}}_{x}{\left({\mathrm{PO}}_{4}\right)}_{6}{\left(\mathrm{OH}\right)}_{2-x}$$

This model also verifies that a decrease in the Ca/P atomic ratio will occur with increasing Ag content in the Ca-deficient AgHAs as a result of Ca depletion from the HA structure upon Ag^+^ incorporation, which is in an agreement with the elemental analysis given in Table 2.

Similarly, weaker FTIR peaks of OH^−^ groups in P2 and P5 compared to those in pure HA can also suggest the incorporation of Ag^+^ ions presumable into Ca(1) sites associated with possible OH^−^ vacancy formations. Moreover, the observation of FTIR peaks belonging to B-type carbonate suggests a possible CO_3_^2−^ incorporation into PO_4_^3−^ sites as well. It should also be observed that an additional decrease in the amount of PO_4_^3−^ due to the possible CO_3_^2−^ anion incorporation will contribute further to the weakening of P–O stretching bands in the P deficient samples.

Normally, monovalent anions can be expected to incorporate directly into OH^−^ positions in the anion channel without causing any charge imbalance. However, divalent anion incorporation of CO_3_^2−^ can be balanced by the formation of both OH^−^ and Ca^2+^ vacancies instead of their direct incorporation into the trivalent PO_4_^3−^ positions [42]. Moreover, the charge imbalance induced by the incorporation of CO_3_^2−^ anions into PO_4_^3−^ sites can also be compensated by oxygen vacancies [43].

Based on these assumptions, the substitution model for the P-deficient AgHAs can be proposed as:

(4) $${\mathrm{Ca}}_{10-x}{\mathrm{Ag}}_{x}\left\{{\left({\mathrm{PO}}_{4}\right)}_{6-y}{\left({\mathrm{CO}}_{3}\right)}_{y}\right\}{\left(\mathrm{OH}\right)}_{2-f\left(x,\;y\right)}$$

**Figure 8:**
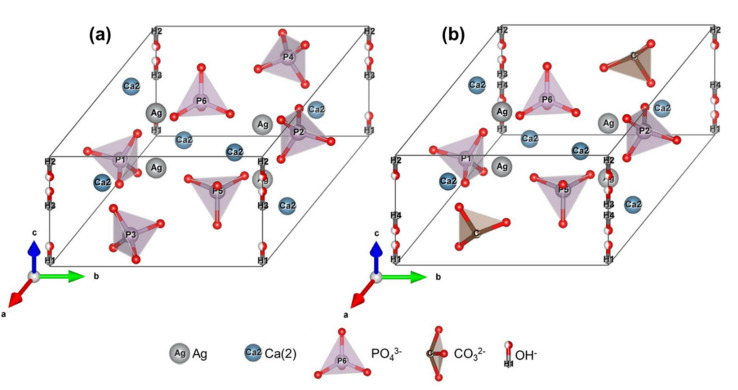
Representations of AgHA structure models synthesized with (**a**) Ca-deficient and (**b**) P-deficient precursors.

The respective illustrations of the proposed models for both Ca- and P-deficient AgHAs are also given in Figure 8, representing Ag^+^ located in the Ca^2+^ position while CO_3_^2−^ is located in the PO_4_^3−^ position specifically in the case of P-deficient AgHA.

Moreover, it is plausible to expect an increasing P depletion due to CO_3_^2−^ incorporation into the PO_4_^3−^ sites contributing to an increase in the Ca/P atomic ratio and also to an increase Ca depletion due to possible Ag^+^ incorporation into Ca^2+^ sites contributing to a decrease in Ca/P atomic ratio with increasing Ag content in the HA structure specifically in the P-deficient AgHAs. EDS analysis (Table 2) verified that there is an overall increase in the Ca/P atomic ratio with an increase in the Ag content suggesting that P depletion has a dominant effect on the Ca/P atomic ratio in the P-deficient AgHAs.

It was reported that an increase in sinterability was observed in HA upon CO_3_^2−^ incorporation particularly into the PO_4_^3−^ sites, which was called B-type carbonated HAs [44,45]. In this respect, the shift in the sintering temperatures into lower temperature regions observed in the P-deficient AgHAs compared to the Ca-deficient ones indicates better sinterability which seems to be due to carbonations in the structure.

### 4.2. The Effect of Variations in the AgHA Structure on In Vitro Behavior

Normally, increasing Ag content in AgHA would increase the antibacterial properties and the toxicity provided by a higher amount of Ag released at a given time. This statement was proven by Rameshbabu et al. [46] with an increase in the amount of Ag exhibiting significant antibacterial activity, however, it also inhibited cell growth. Contrarily, the results in this study clearly showed that the Ag content itself is not the only factor causing differences in antibacterial properties and osteoblast adhesion between the Ca-deficient and the P-deficient AgHAs. Indeed, it seems that structural differences specifically based on the types of structural defects (including the substitutional and interstitial ions as well as associated vacancies) occurred by the facilitation of the differences between the precursor preparation approaches is likely to be a crucial determining factor. Ma et al. reported that carbonated HA synthesized by a co-precipitation method with selenite incorporation using a P-precursor deficient solution precipitation method [33] can be a good choice for improved bone tissue engineering. For instance, facilitated by the introduced weaker Ca–CO_3_ bonds partially replaced with relatively stronger Ca–PO_4_ bonds, the solubility of carbonated HAs can increase [47]. Therefore, it is quite reasonable to expect higher dissolution rates in the P-deficient AgHAs specifically controlled by their carbonate contents than those synthesized from the Ca-deficient precursors. Additionally, the decreased crystallinity of the AgHA samples, as indicated by their broader XRD peaks, should inherently contribute to their dissolution rate compared to pure HA with better crystallinity [47].

Expectedly, C5 showed a higher bactericidal ratio than C2 because of its higher Ag content. In contrast, P5 with higher Ag content unexpectedly had a lower bactericidal ratio than P2. Taking its higher carbonate content into account, it is plausible to expect that P5 should have the highest dissolution rate among the AgHAs. Subsequently, the amount of Ag^+^ ions in the NB solutions should be the greatest dissolved from P5 compared to that of the others tested, and therefore it is expected to have better bactericidal ratios than both P2 and C2. In contrast to this expectation, it presented the lowest value. In fact, the released Ag ions from AgHAs can be removed from the solution by AgCl precipitation as a result of a reaction between the released Ag^+^ and Cl^–^ ions already existing in the NB solutions as reported in our previous studies [13,15,48]. It was also reported that the antibacterial properties of micro-size metallic Ag nanoparticles can remarkably decrease due to the formation of an AgCl layer on their surfaces as a result of a precipitation reaction since AgCl inherently does not have effective bactericidal properties [11,49], although Kim et al. reported the antibacterial effect of AgNO_3_ in spite of AgCl precipitation [50], thus, raising the question of the importance of AgCl size. Another mechanism that prevents the effectiveness of P5 against bacteria might be the high-rate of Ca and P ion dissolution into the bacteria solution, which was expected to reprecipitate as a CaP phase while consuming released Ag^+^ ions from the bacteria solution. On the other hand, Singh et al. observed suppressed Ag^+^ ion release from HA with the presence of a TCP phase [27]. The delayed dissolution mechanism of TCP with Ag incorporation was previously reported [13]. According to these reports, it was assumed that Ca-deficient HA was insufficient to release Ag^+^ ions compared to P-deficient HA.

Therefore, it is quite reasonable to assume that a faster release of Ag ions from P5 facilitated by its higher dissolution rate and fast removal of these highly antibacterial ions by the formation of relatively passive AgCl precipitates in the NB solutions, seems to considerably decrease its antibacterial properties [15]. In contrast, the reason for the interestingly better antibacterial properties of P2, even better than C2, despite lower Ag content should also be related to its relatively higher dissolution rate than C2 due to carbonated AgHA formation. However, its dissolution rate is expected to be relatively lower than that of P5 owing to its lower carbonate content.

In this respect, the time-dependent interaction between Ag^+^ ions and the bacteria in the NB solution seems to be the controlling mechanism for the success of the bactericidal response in AgHAs. If there would be no sufficient time available for the Ag^+^ ions to interact with the bacteria in the NB solution before its removal via the AgCl precipitation, the bactericidal properties can significantly diminish.

Similarly, the lower osteoblast adhesion observed on P2 and P5 even with their lower Ag content compared to their counterparts C2 and C5 seems to be also closely related to their carbonate content and a corresponding increase in dissolution behavior. The increasing dissolution rate of these samples seems to cause an increasing amount of Ag ions released into the testing environment and subsequently showed an increased toxic effect on the osteoblasts. Since P5 had the highest carbonate content, it should have the highest dissolution rate releasing the highest amount of Ag ions/AgCl precipitation, and therefore it showed the highest toxicity among the samples, in which AgCl would prevent cell adhesion due to its effective contact killing properties [13,15].

The present study verified that optimally preventing *E. coli* colonization and assuring sufficient osteoblast adhesion without the use of pharmaceutical drugs, such as antibiotics, which are contributing to the generation of antibiotic-resistant bacteria, can be achieved through Ag incorporated HA. It further demonstrated that in particular P deficiency in the precursor solution, seems to energetically favor the incorporation of CO_3_^2−^ anions into PO_4_^3−^ sites during the synthesis of AgHAs. Eventually, the amount of Ag content at a given time in both of the in vitro test media (specifically, the bacteria and osteoblast media) would mainly be determined by the complicated interaction between the amount of Ag in the sample itself and its dissolution controlled by carbonate content in a particular AgHA, in which P2 samples exhibited the best in vitro performance in this study. If these two parameters can be optimized, the carbonated AgHAs even with a lower Ag content can introduce better bactericidal properties compared to those AgHAs without carbonate content.

Controlling the amount of incorporated CO_3_^2−^ ions provided by P deficiency in the precursor solution can be utilized as an engineering design tool for tailoring time-dependent antibacterial and osteoblast adhesion properties of the synthesized AgHAs for a specific application.

## 5. Conclusions

This study investigated Ag-incorporated HA synthesized from two different precursor solutions in terms of material characterization and in vitro cellular behavior: (a) a Ca-deficient solution with a corresponding ratio of Ca-ions counterbalanced with Ag ions at a stoichiometric value of 1.67 obtained via the (Ca + Ag)/P atomic ratio, (b) a P-deficient solution with a corresponding ratio of P-ions counterbalanced with Ag^+^ ions also at a stoichiometric value of 1.67 obtained via the Ca/(P + Ag) atomic ratio, and (c) one with an extra 0.2 mol Ag added without any compensation in either Ca or P. The results were compared to those of pure HA in terms of material characterization, antibacterial behavior with *E. coli*, and in vitro osteoblast adhesion. The following conclusions can be drawn from the results of this investigation:

1. A higher amount of Ag incorporation was accomplished in the precipitation method when using Ca- or P-deficient precursors compared to extra Ag addition into the precursor. Nevertheless, the highest amount of Ag content was obtained when using Ca-deficient precursors.
2. Ag incorporation occurred into Ca(1) sites associated with an OH^−^ vacancy in both Ca- and P-deficient AgHAs. Additional incorporation of CO_3_^2−^ ions into PO_4_^3−^ sites occurred in the P-deficient AgHAs.
3. Densification started at lower temperatures in the P-deficient AgHA than those of the Ca-deficient ones owing to its carbonated structure.
4. As the Ag content increased in the Ca-deficient AgHAs, the bactericidal properties increased, while osteoblast adhesion decreased.
5. P-deficient AgHAs, even with lower Ag content, exhibited a better combination of bactericidal properties and osteoblast adhesion behavior. On the other hand, both properties substantially diminished as the Ag content increased.

This investigation demonstrated for the first time in the literature that particularly P deficiencies in the precursor solution seemed to energetically favor the incorporation of the CO_3_^2−^ anion into PO_4_^3−^ sites. Eventually, the amount of CO_3_^2−^ ion incorporation controlled by P deficiency can be utilized as a design tool for engineering properties of the synthesized AgHAs for specific applications. Although requiring more investigation, these results indicated that P-deficient AgHA has a great potential as an alternative for bone regeneration with antibacterial properties without resorting to harmful antibiotic use.

## Figures and Tables

**Table 1:** The charged composition and atomic ratios of the Ag-HA series of interest to the present study.

		Charged Molar Ratios			Charged Atomic Ratios			
		Ca	P	Ag	Ca/P	(Ca + Ag)/P	Ca/(P + Ag)	Ag/(Ca + Ag)
Pure HA	HA	10	6	0	1.67	–	–	–
Extra Ag containing HA	E2	10	6	0.2	1.67	–	–	0.0196
Ag ions exchanged with Ca-precursor	C2	9.8	6	0.2	–	1.67	–	0.0200
	C5	9.5	6	0.5	–	1.67	–	0.0500
Ag ions exchanged with P-precursor	P2	10	5.8	0.2	–	–	1.67	0.0196
	P5	10	5.5	0.5	–	–	1.67	0.0476

**Table 2:** Measured composition and atomic ratios of the AgHA series heat treated at 1100 °C and corresponding average grain sizes.

	Intended Ca/P	Measured Atomic Ratios		Concentration Factor (CF)	Average Grain Size (µm)
		Ca/P	Ag/(Ca + Ag)	Ag/(Ag + Ca + P)	
HA	1.67	1.69	–	–	5.8
E2	1.67	2.06	0.0004	0.0003	8.6
C2	1.63	1.99	0.0025	0.0017	5.3
C5	1.58	1.81	0.0061	0.0042	3.3
P2	1.72	1.91	0.0017	0.0011	10.6
P5	1.82	1.99	0.0050	0.0035	6.6

## Data Availability

No new data were created or analyzed in this study.
